# Quick and inexpensive paraffin-embedding method for dynamic bone formation analyses

**DOI:** 10.1038/srep42505

**Published:** 2017-02-15

**Authors:** Amy Porter, Regina Irwin, Josselyn Miller, Daniel J. Horan, Alexander G. Robling, Laura R. McCabe

**Affiliations:** 1 Department of Physiology, Michigan State University, East Lansing, Michigan, USA; 2 Investigative HistoPathology Lab, Michigan State University, East Lansing, Michigan, USA; 3 Department of Anatomy and Cell Biology, Indiana University, Indianapolis, Indiana, USA; 4 Department of Radiology, Michigan State University, East Lansing, Michigan, USA; 5 Biomedical Imaging Research Center, Michigan State University, East Lansing, Michigan, USA

## Abstract

We have developed a straightforward method that uses paraffin-embedded bone for undemineralized thin sectioning, which is amenable to subsequent dynamic bone formation measurements. Bone has stiffer material properties than paraffin, and therefore has hereforto usually been embedded in plastic blocks, cured and sectioned with a tungsten carbide knife to obtain mineralized bone sections for dynamic bone formation measures. This process is expensive and requires special equipment, experienced personnel, and time for the plastic to penetrate the bone and cure. Our method utilizes a novel way to prepare mineralized bone that increases its compliance so that it can be embedded and easily section in paraffin blocks. The approach is simple, quick, and costs less than 10% of the price for plastic embedded bone sections. While not effective for static bone measures, this method allows dynamic bone analyses to be readily performed in laboratories worldwide which might not otherwise have access to traditional (plastic) equipment and expertise.

The skeletal system is highly dynamic and powerful. In addition to providing protection and support for muscles and internal organs, the skeleton is speculated to communicate and regulate the function and health of distant organs such as muscle, pancreas and liver, as well as the endocrine, nervous and immune systems^1^,^2^,^3^,^4^,^5^,^6^,^7^. Correspondingly, studies show that other organs such as brain, muscle, fat, immune cells and the intestine can regulate bone health^6^,^8^,^9^,^10^. Much of the signaling is linked to the function of bone formation by osteoblasts and expression of osteocalcin. Genetically modified mice as well as disease and aging mouse models are utilized to examine factors that regulate osteoblast bone formation and osteocalcin expression. In these studies, accurate measures of bone formation are needed to link signaling pathways or organ function to osteoblast function.

Since the late 1940s, histological examination of undemineralized bone tissue, for a variety of measures including dynamic measures of bone formation, has been facilitated by embedding the bone tissue in one of several plastic formulations^11^. The purpose of plastic embedding for preparing thin sections of undemineralized bone, as opposed to the use of other, more compliant and/or more readily available materials (gelatin or paraffin), is to more closely match the material properties of undemineralized bone with the embedding medium^12^. Because mineralized bone is very stiff, it yields histological sections with less destruction and chatter when the surrounding embedding material is also stiff (e.g., polymerized plastic or hard resins). Those requirements have precluded the use of paraffin—the most widely used embedding medium in histological practice—due to its compliant properties.

The usual solution to this limitation (mismatch of material properties between mineralized bone and paraffin) is to decalcify the bone prior to embedding. This approach works very well and can produce high quality sections, but decalcification of bone prior to sectioning/examination has several drawbacks. One obstacle is the inability to measure dynamic bone formation using *in vivo* incorporation of fluorescent antibiotic derivatives, a technique for quantifying osteoblast activity in vertebrates (including humans) that has been used since the early 1960s^13^. Fluorochrome-derived histomorphometric measurements are still the gold standard for assessing local bone forming activity, but decalcification of the bone tissue removes fluorochrome labeling as well as the mineral component. Consequently, dynamic histomorphometry cannot be performed on decalcified sections.

Thus, the only approach to accurately measure bone formation is to embed and section bone in plastic which requires extensive expertise, specialty equipment, caustic reagents, prolonged plastic infiltration and curing time (typically greater than one week), and hand operated semi-automated microtomes equipped with special tungsten carbide knives to cut the plastic. These significant requirements for plastic embedding remove bone formation analyses as an approach for small labs and/or for research institutes without the specialized equipment and technicians. Here, we developed a method for sectioning mineralized bone that does not require plastic embedding and sectioning. This method can be done with paraffin-based equipment present in a standard histology lab or core.

## Materials and Methods

### Mouse preparation

For dynamic histomorphometric measures of bone formation, calcein (10 mg/kg, Cat no. C0875, Sigma Aldrich, St. Louis, MO) dissolved in 2.0% sodium bicarbonate (pH 7) is filter sterilized and injected intraperitoneally at 7 and 2 days prior to harvest. Injections can be done subcutaneously if preferred. While calcein is commonly used, we have used other fluorochromes, including tetracycline, xylenol orange, and alizarin red complexone among others^14^,^15^. The common doses for fluorochromes ranges from 15 mg/kg to 300 mg/kg^14^,^15^. All experiments were performed in accordance with relevant guidelines and regulations. The Michigan State University Animal Use Committee approved all mouse protocols used.

### Mouse disease model

To compare methods in a disease model, we induced colitis in 5–6 weeks old male mice by providing 1% dextran sodium sulfate (DSS) in their drinking water (MP Biomedicals, 36,000–50,000 MW, Solon, OH). Controls received only water. Mice were housed in a 12-hour light/dark cycle room at 23 °C, allowed to drink and eat (standard chow, Teklad 8640) ad libitum, and were euthanized 15 days after the treatment began.

### Mouse bone harvest

When preparing mouse long bones (i.e., humerus, femur, tibia), the majority of muscle is gently removed from the bone without scraping too close to the bone, which would remove the periosteum. Vertebrae are isolated from approximately L6 to the beginning of the thoracic vertebrae; the latter with its connected ribs provides a land mark. Muscle around the vertebrae is not removed. For dynamic bone analyses, bones are put into plastic 1.5–2.0 ml tubes (or 15 ml tubes for vertebrae) and covered with 10% neutral buffered formalin (NBF). Long bones are typically kept in the fixative for 1 day (if all muscle is removed) up to 3 days at room temperature. The presence of muscle leads to the increased length of time that the long bones are in fixative. Vertebrae (with surrounding muscle intact) are fixed for 1 week. Based on trial and error we found that these incubation times were repeatedly reliable. However, incubation in the fixative for longer than 48 hours can begin to degrade the fluorochrome and reduce signal intensity. Following incubation in NBF the bones are then transferred into 70% ethanol. Overall, a 48 hour formalin fixation period, followed by moving to 50–70% ethanol is the preferred method for achieving both excellent initial cellular fixation, while preserving fluorochrome label.

### Bone processing for paraffin embedding

**Figure 1:**
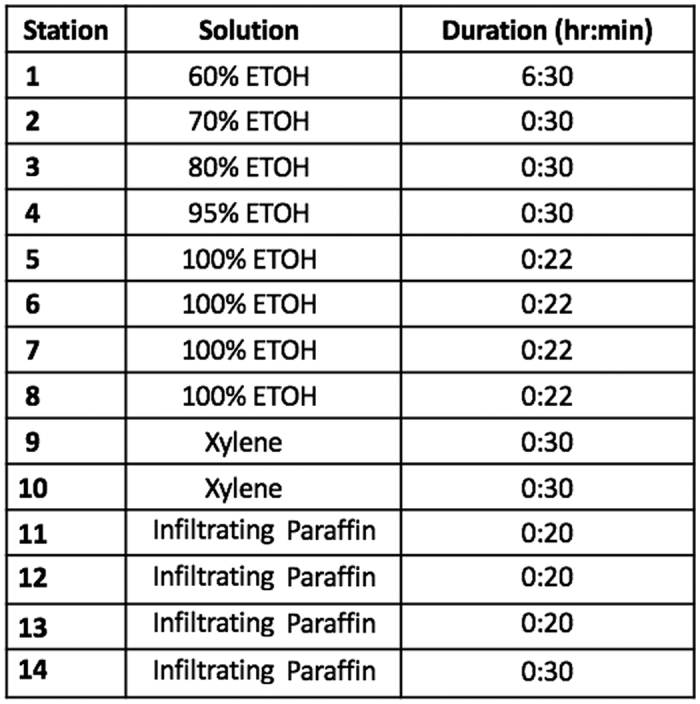
Bone Infiltrating Processing Schedule. After pre-treatment with KOH, bones are placed in histology cassettes and put through the processing sequence outlined above.

Three days prior to processing the bones are placed in individual histology cassettes that are labeled with pencil (or a special solvent resistant marker) and rinsed briefly in distilled water. The size of the mouse bones are approximately 17 × 2 mm for tibias (not shown), 15 × 3 mm for femurs and 3 × 2 mm for a single vertebrae. The cassettes are placed into a specimen container containing 500 mls of 5.0% g/L (10% is also effective) aqueous potassium hydroxide (enough volume to cover ~20 samples and maintain a > 20 ml per sample ratio). Samples are incubated for 96 hours at room temperature with gentle shaking (30 rpm) on an orbital shaker (G-33/MFG: M1071-4000 from New Brunswick, Edison New Jersey). Next, the bones are rinsed by running tap water into the beaker for 2 hours. Once rinsing is complete the samples are kept in 50% ethanol until processed on a vacuum infiltrating tissue processor on the processing schedule described in Fig. 1.

### Bone embedding and sectioning

Processed bones are embedded routinely into paraffin blocks. We embed the vertebrae for transverse sectioning and the long bones for sagittal sectioning; orientation of the bones in this fashion allows for optimal sectioning. Using a Leica RM 2255 semi-automated rotary microtome with a high-grade disposable microtome blade (Duraedge low profile disposable blade), the paraffin blocks are faced at 3 microns by serial sectioning to reach the embedded bone. Once the majority or entire surface of the bone is exposed the block is placed face down into a petri dish containing 1% Aqueous Potassium Hydroxide for 5–10 minutes on a cold plate; a petri dish on ice (roughly 0 °C) will work as well to chill the block. The block is then further cut/faced to obtain a section within the bone. Typically, facing removes about 20 μm of the block. Then final sections, about 6–8 consecutive, full bone sections are obtained. Sections are then taken at 4 microns; placed on charged slides and dried at 56 °C. If more or deeper sections are required, the block can be re-cooled and another 20 μm of the block is removed to get further sections. For bones with less fluorochrome labeling (low bone remodeling), it is helpful to obtain two sections that are ~40 μm apart and get bone measures from both (to increase the bone surface analyzed).

### Vertebrae and long bone dynamic measures

Vertebrae and long bone sections from calcein injected mice are presented randomly and blinded for examination under UV light using a B-2E/C FITC filter (Excitation 465–495 nm) on a Nikon Eclipse E800 microscope (Nikon Instruments Inc, Melville, NY). For the studies noted here, one person read all slides. Five images of the trabecular region (i.e., metaphysis in long bone) and 4 images of cortical regions (from the mid-diaphysis region to the just beneath the growth plate region) are visualized on a computer monitor and images are captured at 20X magnification using a CoolSnap Myo digital camera (Photometrics, Tucson, AZ). Mineral apposition rate (MAR) is analyzed and quantitated digitally from captured digital images. Specifically, MAR is calculated by obtaining the distance between consecutive calcein lines and then dividing by the time of the labeling interval (i.e., for example, in young mice, fluorochrome injections on the mornings of day 7 and day 2 prior to harvest would yield a 5-day labeling interval). The distance between fluorochrome lines is measured utilizing the polygon feature of Image Pro-Plus 7.0 (Media Cybernetics, Rockville, MD) to allow alignment of the polygon along the labeled lines. The average width (space between the two lines) is calculated by dividing the area of the rectangle by the length; this is based on the calculation that the area of a rectangle equals its length X width. Mineralizing Surface (MS) is calculated by measuring the lengths of the bone surface (BS) as well as single label and double label fluorochrome lines using the trace features of Image Pro-Plus 7.0. MS is calculated as (dL+½ sL)/BS where the dL is the length of double labeling, sL is the length of single labeling and BS is the total bone surface. Bone formation rate (BFR) is calculated as BFR = MAR * (MS/BS). Measurements are obtained for each digital photograph of a bone section and are then averaged to yield the measure for one bone. We examined, on average, more than 300 μm of bone surface per bone.

### Statistics

Conditions were compared by 1-way and 2-way ANOVA analyses, as noted, using Prism (GraphPad Software, Inc). Power analyses indicated for comparing two means (ie: control to disease) an n of 6 bones per group was needed for 80% power with a type one error rate of 5% to determine a 25% difference between control and disease groups using either processing method. We were powered to analyze vertebral BFR and long bone BFR and MAR. For vertebrae studies, we removed from our calculations 3 plastic disease bones because of an MAR of 0. The studies were not powered to directly compare differences between paraffin and plastic conditions (ie: control plastic versus control paraffin). For Pearson’s correlation analyses, power analysis (alpha = 0.05, beta = 0.2) indicates an n of 13 pairs is sufficient to detect a correlation of data with an r value greater than 0.7 (UCSF clinical and translation science institute website, http://www.sample-size.net/correlation-sample-size/); the vertebral BFR correlation analyses was significantly powered.

## Results

**Figure 2:**
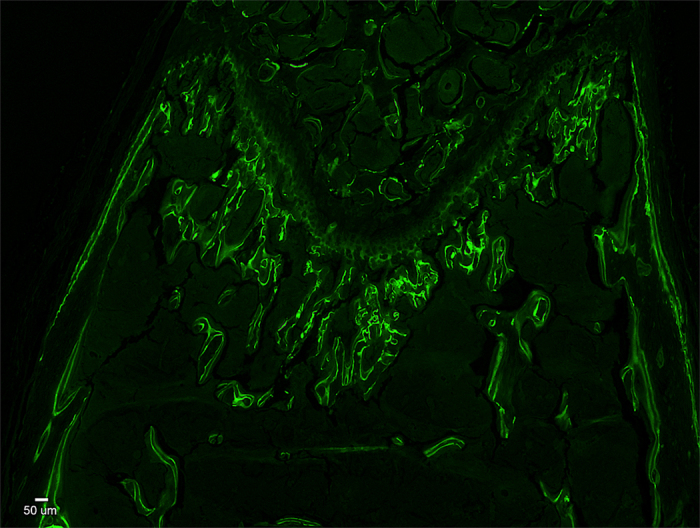
Image of a paraffin embedded mouse femur with calcein fluorochrome labeling of newly synthesized bone. Male mice (8 weeks of age) were injected with 2 pulses of calcein to provide a 5-day labeling interval. The non-decalcified femurs were fixed, processed, embedded in paraffin and 4 μm sections made. Shown is a representative digital image of a sagittal femur section that highlights the distal metaphyseal and epiphyseal trabecular bone formation areas. Cortical bone formation can also be seen on the edge of the bone. Photos were taken at 4X objective power to provide the greatest visual field. The white scale bar is 50 μm in length. The green lines are the fluorochrome incorporated into the newly synthesized bone.

To develop a way that labs across the world could readily section mineralized bone for dynamic bone analyses, we focused on using the paraffin embedding method since this is a straight forward approach that is readily available in nearly every histology laboratory. As noted before, the technical challenge to be overcome, if paraffin is used as an embedding medium, is the difference in stiffness between paraffin and mineralized bone, which prevents it from being effectively sectioned. To overcome this limitation, before bones are processed for dehydration, infiltration and embedding, we pre-incubated the bones in a variety of test solutions to assess their ability to reduce rigidity without demineralizing bone. Solutions tested include: fabric softener (ALL Fabric Softener free/clear for sensitive skin), Nail Soft (Polysciences, Inc.), and a variety of concentrations of potassium hydroxide. Incubations times ranged from 48 to greater than 96 hours (see Table 1). After numerous iterations, we found that incubation of entire femurs (with soft tissue removed manually) in either 5 or 10% KOH was very effective, but with increasing concentrations the marrow integrity of the section was decreased. Thus, we selected 5% aqueous potassium hydroxide for all subsequent studies and incubated the bones in the solution for 96 hours at 30 rpm on a New Brunswick orbital shaker. After this pre-treatment, the bones are rinsed in running tap water and held in 50% ethanol until processed through standard dehydration and paraffin infiltration steps. After embedding the mineralized bone in paraffin, the block is faced in to reach the region of interest. At that point, the block is removed from the microtome chuck and the faced surface is incubated in 1% potassium hydroxide for 5–10 minutes on a cold plate (0 degrees C) prior to the section collection. Through these straightforward steps, we could successfully embed and section mineralized bone in paraffin. The representative mouse femur sagittal section shown in Fig. 2, demonstrate the strong, crisp fluorescent signal of the fluorochrome in the mineralized bone embedded in paraffin. This image is from a 4 μm section.

**Figure 3:**
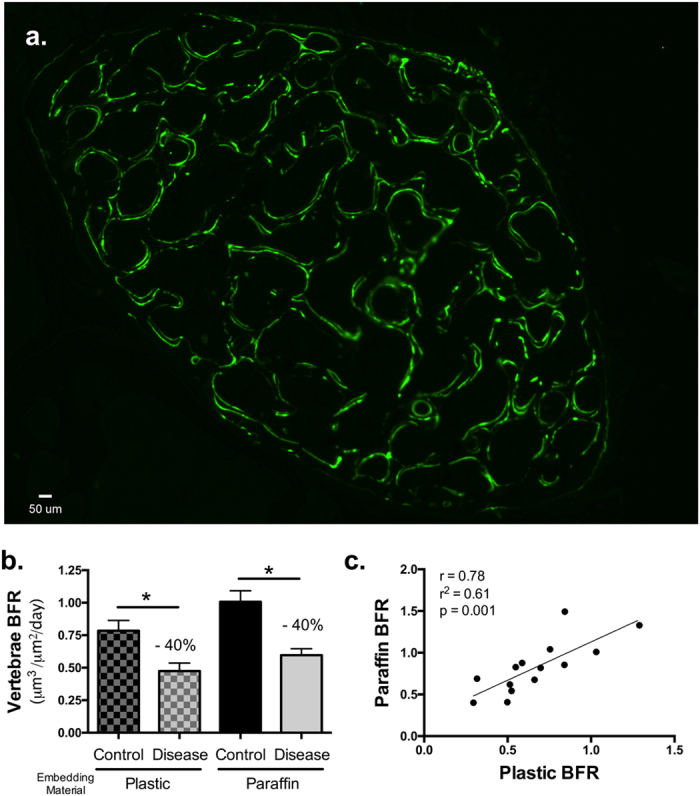
Both plastic and paraffin embedded lumbar vertebrae sections demonstrate reduced trabecular bone formation rate (BFR) in colitis compared to control mice. L3/L4 or L5/L6 vertebrae sets were obtained from all mice and were randomly divided between the standard protocol for plastic processing or the protocol for paraffin processing. Slides were examined at 20X magnification with conditions blinded. (**a**) Representative digital image of a paraffin embedded transverse section of the L3 vertebral body demonstrating regions of trabecular bone formation. Digital images were used to measure vertebral trabecular bone formation rate (BFR, **b**) and bone mineral apposition rate (MAR, **c**). An average of more than 300 μm of bone surface was examined per bone. Pearson’s correlation plots of paraffin compared to plastic BFR (**d**) and MAR (**e**) measures are included; R^2^, r and p values are noted in the plots. Values are averages ± SE. n = 9 for all conditions except for plastic disease which has n = 6 (3 samples had MAR values of 0 and were not included in the data set). *p ≤ 0.05 by one way ANOVA.

To determine whether our method can yield relatively similar results when compared to embedding in plastic, we randomly separated mouse vertebrae (lumbar, L3–4 and L5-6) between processing methods and compared dynamic bone measures between control and diseased mice. For our disease model, we used inflammation-induced bone loss, which consistently demonstrates reduced bone formation^16^. Figure 3a shows, in a representative full transverse section of a paraffin embedded L3 vertebral body, the sharp fluorochrome labeling in the mineralized bone. Vertebral trabecular bone formation rate, BFR was analyzed for control and diseased mouse bones of both processing methods. As expected, the diseased mice have a significant decrease in BFR; this was regardless of the processing method. ANOVA (2-way) did not indicate any interaction between processing and disease conditions, but indicated that disease has a strong impact on BFR (p < 0.0001) and interestingly that the processing condition also had a significant effect (p < 0.01). This is also noted in the Pearson’s correlation analysis where the paraffin embedded bones gave higher values compared to the plastic embedded bones (R^2^ = 0.610, p = 0.001). However, the percent change in BFR in disease mice was − 40% for both groups (calculated based on average BFR for each group: 100 *(C-D)/C), indicating that while BFR may be higher in paraffin sections it is proportionately higher in all groups and does not influence the final outcome of the calculation.

**Figure 4:**
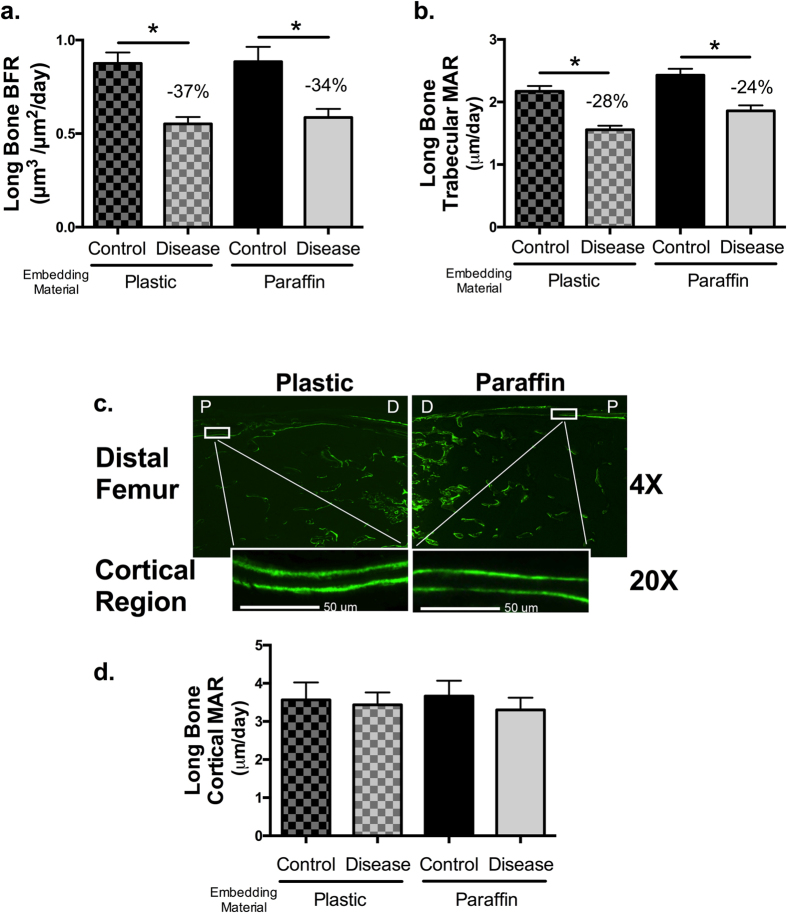
Cortical and trabecular femur MAR and BFR are comparable between plastic and paraffin embedded sections. Whole femurs were obtained from all mice and were randomly divided (left or right) between the standard protocol for plastic processing or the protocol for paraffin processing. Slides were examined at 20X magnification with conditions blinded. (**a**) Long bone formation rate (BFR). (**b**) Long bone trabecular mineral apposition rate (MAR). (**c**) Representative 4X magnification images of fluorochrome bands in mineralized sections of the distal femur embedded in plastic or paraffin showing the metaphyseal and diaphyseal regions. Enlarged images are taken at 20X objective power. The white scale bar is 50 μm in length. (**d**) Long bone cortical MAR. Values are averages ± SE. n ≥ 16 per condition. *p ≤ 0.05 by one way ANOVA.

Next, we tested the method for long bone sectioning and analysis by randomly separating right and left long bones between processing conditions. Examination of the distal femoral metaphyseal bone formation demonstrates clear and defined trabecular surface labeling in the paraffin embedded mineralized bone sections (Figs 2 and 4c). Distal trabecular bone formation rates (Fig. 4a) and mineral apposition rates (Fig. 4b) were similar between the two methods of processing. For long bone, BFR of diseased mouse bones was decreased by 37% for plastic and by 34% for paraffin. For MAR, diseased mouse bones showed a decrease of 28% for plastic and 24% for paraffin. Figure 4c shows images from femora trabecular and cortical regions, but we were also able to use this approach for other long bones, such as the humerus (images not shown). Cortical bone labeling was evident in paraffin sagittal sections, similar to plastic sections. No differences were found in endocortical bone mineral apposition rates between conditions or techniques (Fig. 4c,d).

## Discussion

Increasing reports suggest that the skeletal system may be central to communication between organs, as well as a downstream target of systemic signals. Thus, there is a critical need for assessing bone formation in a broad range of labs since it is required to determine site specific responses to treatments, diseases and genetic modifications. Bone formation rate measures are also often required for research publication to demonstrate an effect of disease or treatment on osteoblast activity *in vivo*. Other ways to address bone formation exist but are not as accurate. For example, serum biomarkers of osteoblast activity such as osteocalcin and procollagen type 1 N-terminal propeptide (P1NP) are commonly used to evaluate bone formation noninvasively, however these measures are incapable of quantifying site-specific local changes in bone formation (ie: cortical versus trabecular bone) due to the total skeletal-averaging effects of serum measurements. More recently, other approaches have been explored, including the use of repetitive micro-computed tomography imaging to track bone volume changes over time^17^, however the repetitive x-ray exposure could impact bone formation rates and therefore only lower resolution (low radiation dose) images can be obtained. Fluorescent imaging technology such as an *in vivo* imaging system, can potentially be used to monitor injected fluorochrome levels in bone to indicate general bone uptake, but the low resolution with this method makes it challenging to measure actual bone formation rates (considering mineral apposition rate and bone surface area). In humans, 18F-NaF (sodium fluoride) positron emission tomography (PET) is also being used as a way to observe bone formation^18^, but again this method does not address specific rate measures and location of bone changes and would not be readily available to the general mouse research lab.

We recognize that there is already an inherent variation in the skeletal system that makes it difficult to demonstrate the precision of any bone analysis methods since similar but different bones must be compared and there is bone-to-bone and mouse-to-mouse variations. Here we focus on the finding that dynamic measures of either plastic or paraffin embedded bones demonstrate that a pathologic condition (intestinal inflammation) suppresses bone formation parameters. Our vertebral BFR analyses indicated that the two methods yield results that are correlated, with the paraffin vertebral sections showing greater fluorescence compared to the plastic as evidenced by 2-way ANOVA indicating a processing effect. This was less apparent for the long bone sections.

It should also be noted that while our method is highly effective at measuring dynamic bone parameters, it is not optimal for static histomorphometry measures. The potassium hydroxide affects bone marrow integrity, especially at higher concentrations (ie: 10%). We are currently testing methods to overcome this effect. Staining of osteoid should be possible with our method, however the staining color observed with stains such as trichrome reagent is somewhat altered.

Taken together, our method is a novel and simple way to process mineralized mouse vertebrae and long bones so that they can be embedded in paraffin and sectioned using standard histology equipment. Given the ease of preparation, this approach can significantly enhance the ability of labs to measure dynamic bone formation rates in their mouse models and opens the door to new discoveries linking osteoblast activity to organ health.

## Additional Information

**How to cite this article:** Porter, A. *et al*. Quick and inexpensive paraffin-embedding method for dynamic bone formation analyses. *Sci. Rep.* **7**, 42505; doi: 10.1038/srep42505 (2017).

**Publisher's note:** Springer Nature remains neutral with regard to jurisdictional claims in published maps and institutional affiliations.

## Figures and Tables

**Table 1:** Pre-treatment solutions tested.

Pre-treatment solution	Incubation time	Bone sectioning results
Fabric Softener #1	96 hour	Could not be sectioned
Fabric Softener #2	96 hour	Could not be sectioned
Nail Soft #1	96 hour	Could not be sectioned
Nail Soft #2	96 hour	Could not be sectioned
2.5% (v/v) Aqueous KOH #1	96 hour	Very poorly at 4 microns
2.5% (v/v) Aqueous KOH #2	96 hour	Very poorly at 4 microns
5.0% (v/v) Aqueous KOH #1	96 hour	Well at 4 microns
5.0% (v/v) Aqueous KOH #2	96 hour	Well at 4 microns
10.0% (v/v) Aqueous KOH #1	96 hour	Very well at 4 microns
10.0% (v/v) Aqueous KOH #2	96 hour	Very well at 4 microns
5.0% (v/v) Aqueous KOH #1	48 hour	Poor at 4 microns
5.0% (v/v) Aqueous KOH #2	48 hour	Poor at 4 microns

For each pilot pre-treatment two bone were tested (#1, #2). Subsequent sectioning information: Fabric Softner: blocks were placed on cold ice block moistened with water for 5 minutes prior to sectioning (no additional softening added); Nail Softener: blocks were placed on cold ice block moistened with water for 5 minutes prior to sectioning (no additional softening added); 2.5% Aqueous potassium hydroxide (KOH): blocks were placed in 1% KOH in a dish on an ice block 10 minutes prior to sectioning at 4 microns – this protocol allowed sectioning of approximately 10 sections before returning to cold plate; 5.0% Aqueous KOH: blocks were placed in 1% KOH in a dish on an ice block 10 minutes prior to sectioning at 4 microns – this allowed us to obtain 6 to 8 sections before returning to cold plate; 10.0% KOH: blocks were placed in 1% KOH in a dish on an ice block 5 minutes prior to sectioning at 4 microns – this allowed us to section approximately 6–8 sections before returning to cold plate; 5.0% KOH for 48 hr – allowed us to obtain 4 sections.
